# Genome-wide analysis of the *GPAT* gene family in wheat and the potential roles of *TaGPAT58* and its homologs in male reproductive development

**DOI:** 10.3389/fpls.2026.1779919

**Published:** 2026-02-27

**Authors:** Hao Zhou, Yingkun Wang, Fujing Yang, Zhiwei Sun, Liwen Meng, Zhangpeng Shi, Na Niu, Lingjian Ma

**Affiliations:** College of Agronomy, Northwest A&F University, Yangling, Shaanxi, China

**Keywords:** expression patterns, *GPAT* gene family, male reproductive development, *TaGPAT58*, wheat

## Abstract

The glycerol-3-phosphate acyltransferase (GPAT) gene family plays a critical role in the biosynthesis of lipids in plants. However, the *GPAT* gene family has not yet been systematically analyzed in wheat, and in particular, the relationship between the *GPAT* genes and male fertility in wheat (*Triticum aestivum* L.) remains unclear. In this study, a total of 64 *TaGPAT* genes were identified at the whole-genome level and classified into three clades. The genes within each clade exhibited conserved motif distributions and gene structures, whereas clear differences were observed among the different clades. A synteny analysis indicated that segmental duplication was the major driving force for the expansion of the *TaGPAT* gene family. An analysis of the expression pattern showed that the *TaGPAT* genes showed distinct expression patterns among different tissues and reproductive stages, with some genes preferentially expressed in roots, grains, or spikes, and specific *TaGPAT* genes reaching peak expression at key meiotic stages. In the temperature-sensitive male-sterile wheat line YS3038, *TaGPAT58* was specifically highly expressed at the trinucleate pollen stage under fertile conditions, and the encoded protein was localized to the endoplasmic reticulum. Virus-induced gene silencing targeted to *TaGPAT58* resulted in a reduction in the number of pollen, abnormal pollen morphology, and a significant decrease in the seed-setting rate. Collectively, this study provides a comprehensive characterization of the *TaGPAT* gene family in wheat and evidence that *TaGPAT58* and its two homoeologous genes are involved in male reproductive development, thereby offering important insights into the molecular mechanisms of male sterility and the exploitation of heterosis in wheat.

## 1 Introduction

Wheat (*Triticum aestivum* L.) is one of the most important grain crops throughout the world (Ray et al., 2013), and hybrid breeding is considered an effective approach to increase the yield of grain per unit area and improve agronomic traits in wheat (Schnable and Springer, 2013). However, because wheat is a predominantly self-pollinated crop, the effective exploitation of heterosis remains challenging (Farooq et al., 2024; Longin et al., 2012). One of the key prerequisites to implement hybrid breeding in wheat is the establishment of stable and efficient male-sterility systems that aim to suppress self-pollination, while enabling the cross-pollination of female gametes with selected male parents (Revell et al., 2025). Therefore, elucidating the molecular mechanisms underlying male sterility is critical for promoting the efficient utilization of heterosis in wheat.

The metabolism of lipids plays a crucial role in the reproductive development of plants, particularly in the development of the anther cuticle and pollen wall (Shi et al., 2015). The anther cuticle and pollen exine are two layers that are rich in lipids and essential for the fertility of pollen (Zhang et al., 2016). The anther cuticle is an extracellular lipid layer that covers the surface of anthers and protects the anther from abiotic stresses, prevents the dehydration of internal tissues, and defends against infection by pathogens (Kunst and Samuels, 2009). Glycerol-3-phosphate acyltransferase (GPAT, EC 2.3.1.15) is a key enzyme that catalyzes the initial step in the biosynthesis of glycerolipids (Gidda et al., 2009; Singer et al., 2016). It can be classified into *sn*-1 and *sn*-2 types. The *sn*-1 type GPAT transfers acyl groups from acyl-CoA or the acyl-acyl carrier protein (acyl-ACP) to the *sn*-1 position of glycerol-3-phosphate, which forms lysophosphatidic acid (LPA). It primarily participates in the biosynthesis of membrane lipids and storage lipids. The *sn*-2 type GPAT retains phosphatase activity and primarily produces 2-monoacylglycerol (2-MAG), which is primarily involved in the biosynthesis of cutin and suberin (Beisson et al., 2012; Li et al., 2007b; Yang et al., 2010) and play an important role in anther cuticle formation (Wan et al., 2020). Subcellular localization enables the categorization of GPATs into plastidial, endoplasmic reticulum (ER), and mitochondrial types. Each plays an important biological role during the development of plants (Chen et al., 2011). The plastidial GPATs, represented by AtS1 in *Arabidopsis thaliana* (*A. thaliana*), catalyze the first step in the assembly of glycerolipids by transferring acyl groups to glycerol-3-phosphate (G3P) to generate lysophosphatidic acid (LPA), a key intermediate in lipid metabolism (Kang et al., 2020). The GPATs that are localized in the ER are primarily involved in the biosynthesis of lipid polyesters, such as cutin and suberin (Li et al., 2007a). Additionally, since the biosynthesis of triacylglycerol and membrane lipids predominantly occurs in the ER, this type of GPAT is also considered to participate in the biosynthesis of these lipids (Gidda et al., 2009). *OsGPAT03* is localized to the ER and indispensable for pollen development in rice. The *osgpat03* mutant has smaller anthers, defective anther cuticles and pollen exines, and markedly reduced levels of aliphatic lipids in the anthers (Men et al., 2017). Mitochondrial GPATs are closely associated with the development of pollen and also contribute to the biosynthesis of storage lipids (Zheng et al., 2003). It has been shown that mutations in mitochondrial *GPAT* can lead to excessive fragmentation of the mitochondria (Ohba et al., 2013). Therefore, a systematic analysis of the composition of the *GPAT* gene family and its roles in anther and pollen development is crucial for a deeper understanding of the lipid metabolic regulation underlying male fertility in plants.

The *GPAT* gene family has been systematically identified in a variety of plant species, including *A. thaliana*, barley (*Hordeum vulgare*), rice (*Oryza sativa*), maize (*Zea mays*), and alfalfa (*Medicago sativa*) (Ma et al., 2024; Safder et al., 2021; Waschburger et al., 2018; Yang et al., 2024; Zhu et al., 2019). Previous studies have reported that *GPAT* genes are involved in male fertility by regulating anther and pollen development in both model plants and major crops, as summarized below. In *A. thaliana*, the loss of function of *AtGPAT01* leads to the severe arrest of pollen development, and the complementation of the mutant restores the phenotype, thus, confirming the critical role of *AtGPAT01* in pollen development (Zheng et al., 2003). *AtGPAT09* is highly conserved throughout evolution and predominantly exists as a single copy in most plants. The protein encoded by *AtGPAT09* localizes to the ER, and knockout mutants in *A. thaliana* exhibit gametophytic lethality, while the seed-specific knockdown results in a significant reduction in the content of seed oil (Shockey et al., 2016). Scanning electron microscopy and transmission electron microscopy analyses of the *atgpat6–3* mutant further demonstrated that the mutant pollen grains were collapsed and possessed irregular pollen walls. There was severe suppression of the expansion of ER in the tapetal cell, and there were defects in the accumulation of lipid bodies and deposition of a coating on the pollen wall. These effects indicate that *GPAT6* plays a crucial role in pollen development, particularly in the formation of pollen walls and accumulation of storage material (Li et al., 2012). In maize, developmental differences between the *zmgpat6* mutant and wild type begin at stage S10. At this stage, the wild type anther cuticle gradually develops a three-dimensional reticulate structure, whereas the surface of mutant anthers remains smooth and fails to form such structures even at the final developmental stage. Moreover, starting from S10, Ubisch bodies outside the tapetum gradually disappear in the wild type but remain visible in the mutant. In terms of pollen development, the mutant is defective in its formation of pollen exine, which ultimately leads to dysfunctional pollen (Zhu et al., 2019). However, the *GPAT* gene family in wheat has not yet been systematically characterized, and its relationship with male fertility remains unexplored.

In this study, 64 *TaGPATs* were identified using bioinformatic approaches. To further elucidate the evolutionary history of the wheat *GPAT* gene family, we also identified 21, 44, 41, and 17 *GPAT* genes in *Aegilops tauschii* (*Ae. tauschii*), *Triticum dicoccoides* (*T. dicoccoides*), *Triticum turgidum* (*T. turgidum*), and *Triticum urartu* (*T. urartu*), respectively. Phylogenetic and gene duplication analyses along with the characterization of gene structure were subsequently performed to elucidate the evolutionary history and structural features of the *TaGPATs*. In addition, promoter and GO enrichment analyses and expression profiling were conducted to further explore their potential biological functions. Finally, to better clarify the role of *TaGPAT58* and its two homoeologous genes in the development of pollen in wheat, its function was validated through subcellular localization analysis and virus-induced gene silencing (VIGS).

## 2 Materials and methods

### 2.1 Identification of *GPAT* gene family members in wheat and its progenitor species

Genome data of wheat and its progenitor species were retrieved from Ensembl Plants (https://plants.ensembl.org). The reference genome of wheat was from release 61 (IWGSC RefSeq v1.0), and the genomes of *Ae. tauschii* (Aet v4.0), *T. dicoccoides* (WEWSeq v1.0), *T. turgidum* (Svevo v1), and *T. urartu* (Tu2.0) were from release 62 of the same database. The Hidden Markov Model (HMM) profile of the acyltransferase domain (PF01553) was obtained from the InterPro database (https://www.ebi.ac.uk/interpro/). Proteins that contained this domain were identified in the local wheat protein database using HMMER 3.3.2, with an E-value threshold of 1.0E^-5^ (Lei et al., 2021). Subsequently, the GPAT protein sequences from rice (Safder et al., 2021) and *A. thaliana* that were obtained from the TAIR database (https://www.arabidopsis.org/) were used as queries. BLASTP searches were performed against the local wheat protein database with an E-value threshold of 1.0E^-5^. Protein sequences obtained from both methods were integrated and manually deduplicated, and they yielded candidate members of the wheat *GPAT* gene family. These candidates were submitted to InterPro (Finn et al., 2017) and NCBI-CDD to confirm the presence of the PlsC acyltransferase domain, thereby determining the final *GPAT* gene family members. Additionally, the *GPAT* gene family members in the following progenitor species of wheat: *Ae. tauschii*, *T. dicoccoides*, *T. turgidum*, and *T. urartu* were identified using the same methods. The chromosomal physical localization information was sourced from the wheat genome annotation data, and MG2C was employed to map the wheat *GPAT* gene family members to chromosomes (Chao et al., 2021). The molecular weight (MW) and isoelectric point (pI) of the wheat GPAT family proteins were calculated using the pepstats module of EMBOSS 6.6.0 with default parameters (Rice et al., 2000). Subcellular localization of the family members was predicted using the online tool DeepLoc 2.1 (https://services.healthtech.dtu.dk/services/DeepLoc-2.1/).

### 2.2 Phylogenetic relationship, gene structure, and conserved motif analysis

To investigate the evolutionary relationships of the wheat *GPAT* gene family, GPAT protein sequences from wheat, maize, *A. thaliana*, *Ae. tauschii*, *T. dicoccoides*, *T. turgidum*, and *T. urartu* were aligned using the ClustalW algorithm implemented in MEGA11 (Tamura et al., 2021) with default parameters, and a neighbor-joining (NJ) phylogenetic tree was subsequently constructed in MEGA11 with 1,000 bootstrap replicates, while all other parameters were set to their default values. The phylogenetic tree was visualized using Evolview (He et al., 2016).

Information about the gene structures of *TaGPATs* was extracted from genome annotation files to analyze the patterns of distribution of the introns and exons. Conserved motifs in the TaGPAT protein sequences were identified using MEME 5.5.2 (Bailey et al., 2015) with default parameters and a maximum motif number set to 20, and the predicted motifs were subsequently analyzed for functional annotation using the InterPro database. The results were visualized using TBtools (Chen et al., 2023).

### 2.3 Gene duplication and synteny analysis

The evolutionary relationships and syntenic characteristics of the *GPAT* gene family in wheat and its related species were elucidated by conducting gene duplication and synteny analyses among wheat, *Ae. tauschii*, *T. dicoccoides*, *T. turgidum*, *T. urartu*, and rice. MCScanX (Wang et al., 2012) was used for the synteny analysis with default parameters. Circos 0.69.9 (Krzywinski et al., 2009) was used for visualization. The CDS and protein sequences of the syntenic gene pairs were aligned using ParaAT 2.0 (Zhang et al., 2012), and the nonsynonymous substitution rate (Ka) and synonymous substitution rate (Ks) were calculated then using the KaKs_Calculator 3.0 (Zhang, 2022), both with default parameters. Finally, the divergence time (T) of the syntenic gene pairs was estimated using the formula, with λ = 6.5 × 10^-9^ for *Gramineae* (Gaut et al., 1996).


$$\text{T}=\text{Ks}/(2\text{λ})\times 10^{6}\text{ Mya}$$

### 2.4 Promoter analysis of the *TaGPATs*

The 2,000 bp upstream sequences from the translation start site (ATG) of *TaGPAT* genes were retrieved from the Ensembl Plants database and submitted to the PlantCARE database (https://bioinformatics.psb.ugent.be/webtools/plantcare/html/) to predict *cis*-acting regulatory elements in the promoter regions.

### 2.5 Analysis of the gene ontology annotation

All 64 *TaGPAT* genes were submitted to the TGT database (https://wheat.cau.edu.cn/TGT/) for the GO annotation analysis, and the results were visualized using R.

### 2.6 Analysis of tissue expression patterns

The WheatOmics database (Ma et al., 2021) was the source of the transcriptome data of *TaGPATs* in five tissues (roots, stems, leaves, spikes, and grain) and at four meiotic stages, including the latent leptotene to leptotene (latent_lepto), diplotene to diakinesis (diplo_dia), zygotene to pachytene (zygo_pachy), and metaphase I. Expression heatmaps were constructed using GraphPad Prism 10 (Dotmatics, Boston, MA, USA).

### 2.7 Plant materials and qRT-PCR analysis

The relationship between the *TaGPATs* and pollen fertility in wheat was explored using the temperature-sensitive male sterile line YS3038, which was created in our laboratory, as the experimental material. The male fertility of this line is regulated by temperature but unaffected by the photoperiod (Ma et al., 2022). The plants were subjected to a low-temperature environment (17/13 °C) 1 week prior to meiosis, which resulted in sterile pollen, which was designated as YSS. Conversely, fertility was restored to the plants under a higher temperature regime (24/20 °C), and they were designated YSF. The anthers at the three developmental stages uninucleate (U), binucleate (B), and trinucleate (T) were collected for total RNA extraction. The total RNA was isolated using the RNAiso Reagent (TaKaRa, Beijing, China) with three biological replicates. The cDNA was synthesized using the Evo M-MLV RT Mix Kit with gDNA Clean for PCR (Accurate Biology, Changsha, China). Quantitative real-time PCR (qRT-PCR) was performed using the QuantStudio™ 7 Flex Real-Time PCR System (Thermo Fisher Scientific, Waltham, MA, USA), and the levels of relative gene expression were calculated using the 2^-ΔΔCt^ method (Livak and Schmittgen, 2001) with *TaACTIN* as the reference gene (Paolacci et al., 2009). The primers were designed using Oligo 7, and their sequences are listed in **Supplementary Table 14**.

### 2.8 Subcellular localization of *TaGPAT58*

The subcellular localization of TaGPAT58 was determined by transient expression in wheat protoplasts. Approximately 2 g of seedlings that were grown in the dark at 24 °C for approximately 10 days were finely chopped and incubated in an enzyme solution (1.5% Cellulase R10, 0.4% Macerozyme R10, 0.4 M mannitol, 20 mM potassium chloride [KCl], and 10 mM MES [(pH 5.7)]) at 24 °C for 4 h. After the macerated tissue was filtered through a mesh (40 μm), the suspension was centrifuged at 400 rpm for 3 min, and the supernatant was discarded. The pellet was washed twice with 10 mL pre-chilled W5 solution (154 mM sodium chloride [NaCl], 125 mM calcium chloride [CaCl_2_], 2 mM potassium dihydrogen phosphate [KH_2_PO_4_], 2 mM MES, and 5 mM glucose) and centrifuged at 300 rpm and 4 °C for 3 min. The supernatant was removed. The pellet was then resuspended in an appropriate volume of MMG solution (0.4 M mannitol, 15 mM magnesium chloride hexahydrate [MgCl_2_·6H_2_O], and 4 mM MES) as required (Yoo et al., 2007).

The target protein was expressed by cloning the *TaGPAT58* coding sequence without the stop codon into the pCAMBIA1302-EGFP vector (restriction enzyme: BstBI). The primer information is listed in **Supplementary Table 14**. Transformation mediated by PEG was used to introduce the recombinant plasmid into the wheat protoplasts. Briefly, 200 μL of the protoplast suspension was mixed with 10 μL of the target gene plasmid and 10 μL of an ER marker plasmid, followed by the addition of an equal volume (220 μL) of PEG4000. The mixture was gently mixed and incubated at room temperature for 10–15 min. The reaction was stopped by dilution with 1 mL of W5 solution. The protoplasts were collected by centrifugation at 400 rpm for 3 min, and the supernatant was discarded. The pellet was washed once or twice with 1 mL of W5 solution. Finally, the protoplasts were resuspended in 1 mL of W5 solution and incubated under light at 24 °C for 18–24 h. After centrifugation and the removal of most of the supernatant, approximately 100 μL of protoplasts was retained for observation using laser scanning confocal microscopy.

### 2.9 Silencing of *TaGPAT58* by BSMV-VIGS and the phenotypic analysis

Barley stripe mosaic virus-mediated gene silencing (BSMV-VIGS) was employed to silence *TaGPAT58* in wheat anthers to validate its role in male fertility as previously described (Han et al., 2021). Four vectors, including α, β, γ, and γ-phytoene desaturase (PDS), were used in this study. A 270 bp fragment of *TaGPAT58*, designed to be gene-specific, except for its high similarity to the paralogs *TaGPAT55* and *TaGPAT56*, was cloned into the γ vector by homologous recombination. The restriction enzyme MluI was used to linearize the α and γ plasmids; SpeI was used to linearize the β plasmid, and BssHII was employed to linearize both the γ-*TaGPAT58* and γ-*PDS* plasmids. The RiboMAX™ Large Scale RNA Production System-T7 (Promega, Madison, WI, USA) was used for the *in vitro* transcription of the linearized plasmids. The wheat line YS3038 was grown under fertile conditions until the flag leaf had fully emerged. *In vitro* transcribed RNAs from each of the three plasmids per combination (0.5 μL each) were mixed with 9 μL GK-P buffer (50 mM glycine, 1% bentonite, 30 mM K_2_HPO_4_, and 1% diatomaceous earth) to prepare the inoculation solution. Each inoculation solution was applied to one flag leaf, and 10 plants were treated per group. GK-P buffer alone served as the mock control; the α, β, and γ vector combination served as the negative control; the α, β, and γ-PDS combination served as the positive control, and the α, β, and γ-*TaGPAT58* combination served as the treatment group. Phenotypic changes in the leaves were observed 14 days post-inoculation. The anthers were collected at the trinucleate stage and crushed. They are then stained with a solution I_2_–KI to evaluate the pollen fertility. Pollen grains that were uniformly stained dark black and exhibited a plump, near-spherical morphology were classified as normal pollen, whereas pollen grains that weakly stained as shown by their yellow–brown coloration and irregular or shriveled morphology were classified as abnormal pollen. Subsequently, a qRT-PCR analysis was performed to determine the silencing efficiency of the target gene. Finally, the seed setting rates of the negative control and treatment groups were analyzed statistically. The primer and sequence information are provided in the **Supplementary Table 14**.

### 2.10 Statistical analysis

All experiments were performed with three independent biological replicates. The data are presented as the mean ± SD. Statistical significance between the groups was assessed using Student’s *t*-test, with ** indicating *p* ≤ 0.01.

## 3 Results

### 3.1 Genome-wide identification of the *GPAT* genes in wheat and its four progenitor species

*GPAT* genes in wheat were identified using the acyltransferase domain (PF01553) HMM with HMMER, combined with BlastP searches using rice and *A. thaliana* GPAT proteins as queries. Candidate sequences were further confirmed with InterPro and NCBI CDD to ensure the presence of the PlsC acyltransferase domain. Using this approach, 64 *GPAT* genes were identified in wheat. The same strategy was applied to wheat progenitors, resulting in the identification of 21, 44, 41, and 17 *GPAT* genes in *Ae. tauschii*, *T. dicoccoides*, *T. turgidum*, and *T. urartu*, respectively. The candidates were validated using InterPro and NCBI-CDD to confirm the conserved domains. All the genes were named based on their chromosomal locations within each species as follows: *TaGPAT01*–*TaGPAT64*, *AetGPAT01*–*AetGPAT21, TdGPAT01–TdGPAT44, TtGPAT01–TtGPAT41*, and *TuGPAT01–TuGPAT17*. Chromosomal mapping revealed that 62 *TaGPAT* genes were unevenly distributed across all 21 wheat chromosomes, whereas two genes (*TaGPAT63* and T*aGPAT64*) were located on unassembled scaffolds. Among them, 22, 21, and 19 *TaGPATs* were located in the A, B, and D subgenomes, respectively. In addition, 49 *TaGPATs* were distributed on chromosome groups 1–4, while only 13 genes were located on chromosome groups 5–7 (**Supplementary Figure 1**).

The subcellular localization of 64 TaGPAT proteins was predicted using the DeepLoc 2.1 online server, and the MW and pI were calculated using the pepstats module in EMBOSS (**Supplementary Table 1**). All the TaGPAT proteins exhibited conserved subcellular localizations, with 59 localized to the endoplasmic reticulum (ER), four to the plastid, one to the mitochondrion or ER, respectively. The TaGPAT proteins ranged from 364 to 587 amino acids long with an average of 498 amino acids, and their MW ranged from 42.13 to 64.42 kDa.

### 3.2 Phylogenetic and evolutionary analysis of the *GPAT* gene family in wheat

**Figure 1:**
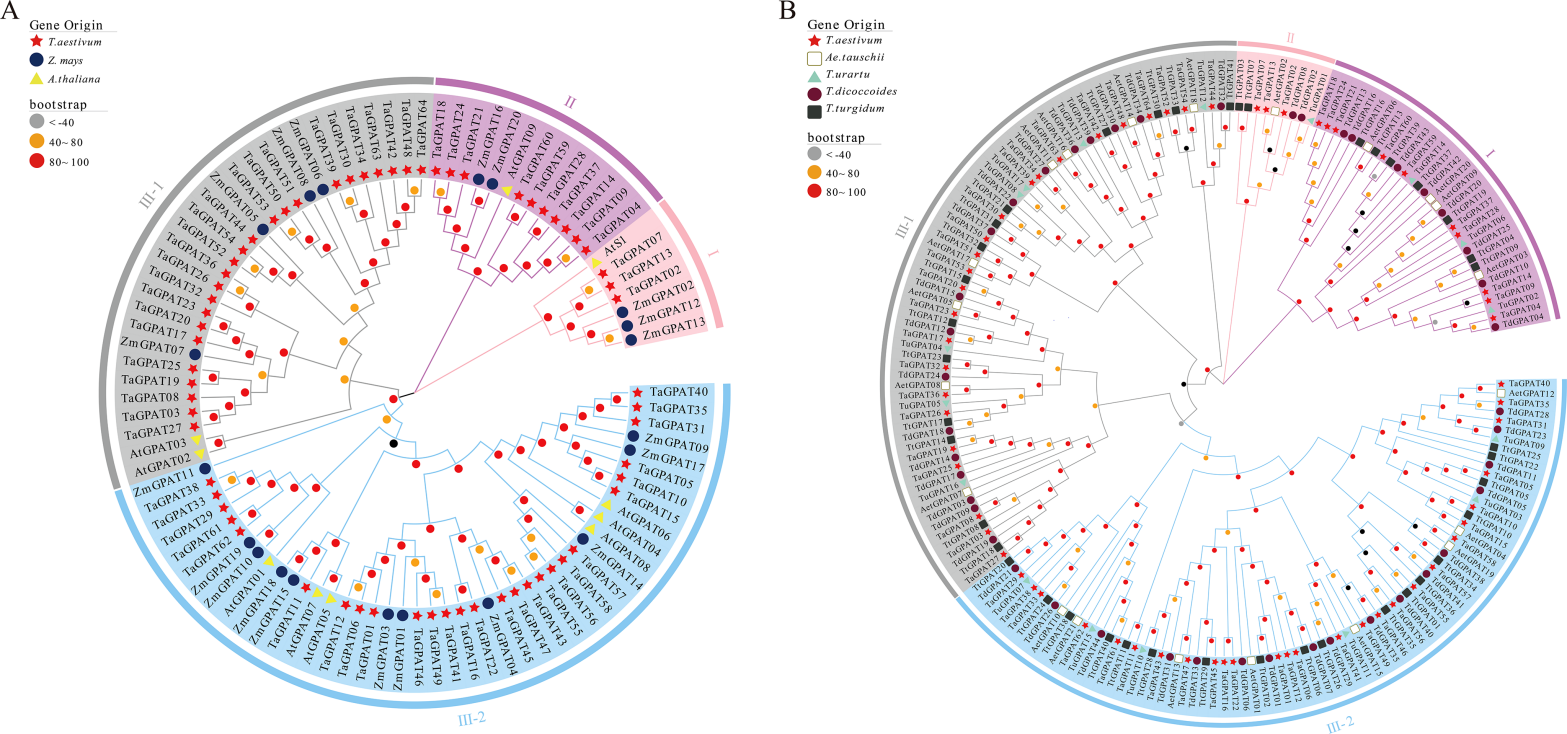
Phylogenetic analysis of the GPAT gene family in wheat and related species. **(A)** Phylogenetic tree constructed using the full-length GPAT protein sequences from *Triticum aestivum*, *A. thaliana*, and *Zea mays*. **(B)** Phylogenetic relationships of GPAT genes between wheat and its progenitor species, including *Ae. tauschii*, *T. dicoccoides*, *T. turgidum*, and *T. urartu*. The trees were generated using the neighbor-joining method with 1,000 bootstrap replicates. Different colored clades indicate distinct evolutionary groups.

To evaluate the evolutionary relationships of the *GPAT* genes in wheat, maize, and *A. thaliana*, a neighbor-joining (NJ) phylogenetic tree was constructed based on the multiple sequence alignment of 64 TaGPAT, 10 AtGPAT, and 20 ZmGPAT full-length protein sequences (**Figure 1A**). The 94 genes were grouped into three major clades, and Clade III was further subdivided into two subclades. Each clade contained genes from all three species. *TaGPAT* genes from the A, B, and D subgenomes are closely grouped together, reflecting their orthologous relationships among the three subgenomes. Additionally, 30 pairs of paralogous genes were identified within the NJ tree, including five pairs from maize, three pairs from *A. thaliana*, and 22 pairs from wheat. Considering that common wheat is a hexaploid species, its genome naturally harbors a higher number of homoeologous copies. Clade III-2 contains *TaGPAT58* and its two homologs, *TaGPAT55* and *TaGPAT56*, together with *AtGPAT04*, *AtGPAT06*, and *AtGPAT08*. The evolutionary relationships between these three *TaGPAT* genes and the three *AtGPAT* genes provide clues for functional studies of *TaGPAT58* and its homologs.

The same approach was used to construct phylogenetic trees for wheat and its four progenitor species (**Figure 1B**). A total of 187 genes were grouped into three clades, and Clade III contained the largest number of members. We identified 11 orthologous gene pairs between wheat and *Ae. tauschii*, 14 orthologous pairs between wheat and *T. turgidum*, and six orthologous pairs between wheat and *T. dicoccoides*. However, no orthologous pairs were found between wheat and *T. urartu*.

### 3.3 Analyses of the conserved motifs and gene structure of the *TaGPATs*

**Figure 2:**
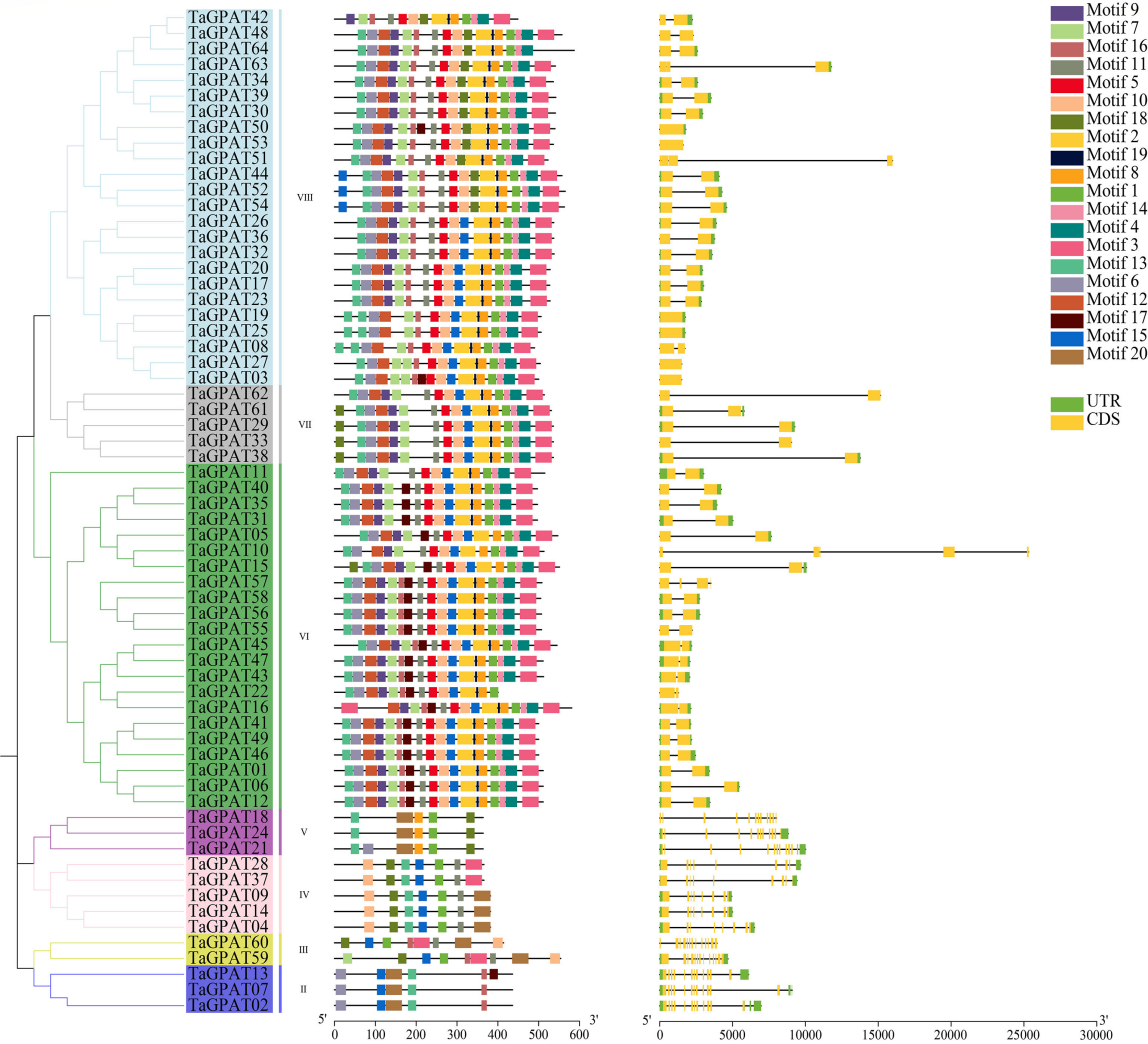
Phylogenetic relationships, conserved motifs, and gene structures of *TaGPATs*. The phylogenetic tree of TaGPAT proteins is shown on the left. The conserved motifs of TaGPAT proteins identified by MEME are displayed in the middle, with different colored boxes representing different motifs. The gene structures are shown on the right, where yellow boxes indicate CDS, black lines represent introns, and green boxes denote untranslated regions (UTRs).

Motif structures encoded by conserved genes and exon–intron organization represent important evolutionary signatures. The MEME web server was employed to identify conserved motifs among the 64 members of the *TaGPAT* gene family. A total of 20 distinct conserved motifs were detected and designated motif 1–motif 20 (**Figure 2**; **Supplementary Table 2**). Each motif was annotated by searching the InterPro database. A phylogenetic tree of the *TaGPATs* was constructed to elucidate the structural conservation of *TaGPATs* by dividing them into three clades (**Figure 2**). Overall, the members within the same clade exhibited similar conserved motifs and gene structures, whereas significant differences were observed among the motif number, arrangement, and exon count in the different clades. This indicates notable structural divergence within the gene family.

Among the 20 conserved motifs, motif 2 is associated with Glycerol-3-phosphate-acyltransferase, and motif 6 and 7 are associated with Glycerol-3-phosphate acyltransferase RAM2-like/HAD-like domains. However, twelve motifs did not match any known functional domains in the database, suggesting that they may represent novel motifs within the *TaGPAT* family. Motif distribution across clades revealed both conserved and clade-specific patterns. Motif 2 was specific to Clade III, motif 6 and 7 were present in over 80% of the *TaGPAT* members, indicating that these motifs are highly conserved within the family and likely play core functional roles. Motif 6, 13, 15, and 16 are shared across all three clades (I, II, III), indicating their conserved roles in *TaGPAT* structure or function. Several motifs exhibit clade-specificity: motif 2, 4, 5, 9, 12, 14, and 19 are unique to Clade III.

Moreover, substantial variation exists among the clades in terms of the size of clade and complexity of motif. Clade III is the largest and contains 51 members. Each possessed more than 14 motifs. In contrast, Clades I and II contained fewer members (3 and 10, respectively), and each gene only harbored five to nine motifs. The exon–intron structures further corroborated this pattern of differentiation. The genes in Clade III are highly streamlined and generally only contain one to two exons. Conversely, the genes in Clades I and II display more complex and conserved exon architectures, which demonstrate their clear specific characteristics per clade. Together, these findings suggest that the *TaGPAT* gene family has substantially diverged in structure during evolution. The highly conserved motifs maintain the fundamental biochemical activities of the family, while the pronounced differences in gene structure and motif composition among clades drive the functional diversity of the *TaGPATs*.

### 3.4 *TaGPATs* gene duplication events and synteny analysis

**Figure 3:**
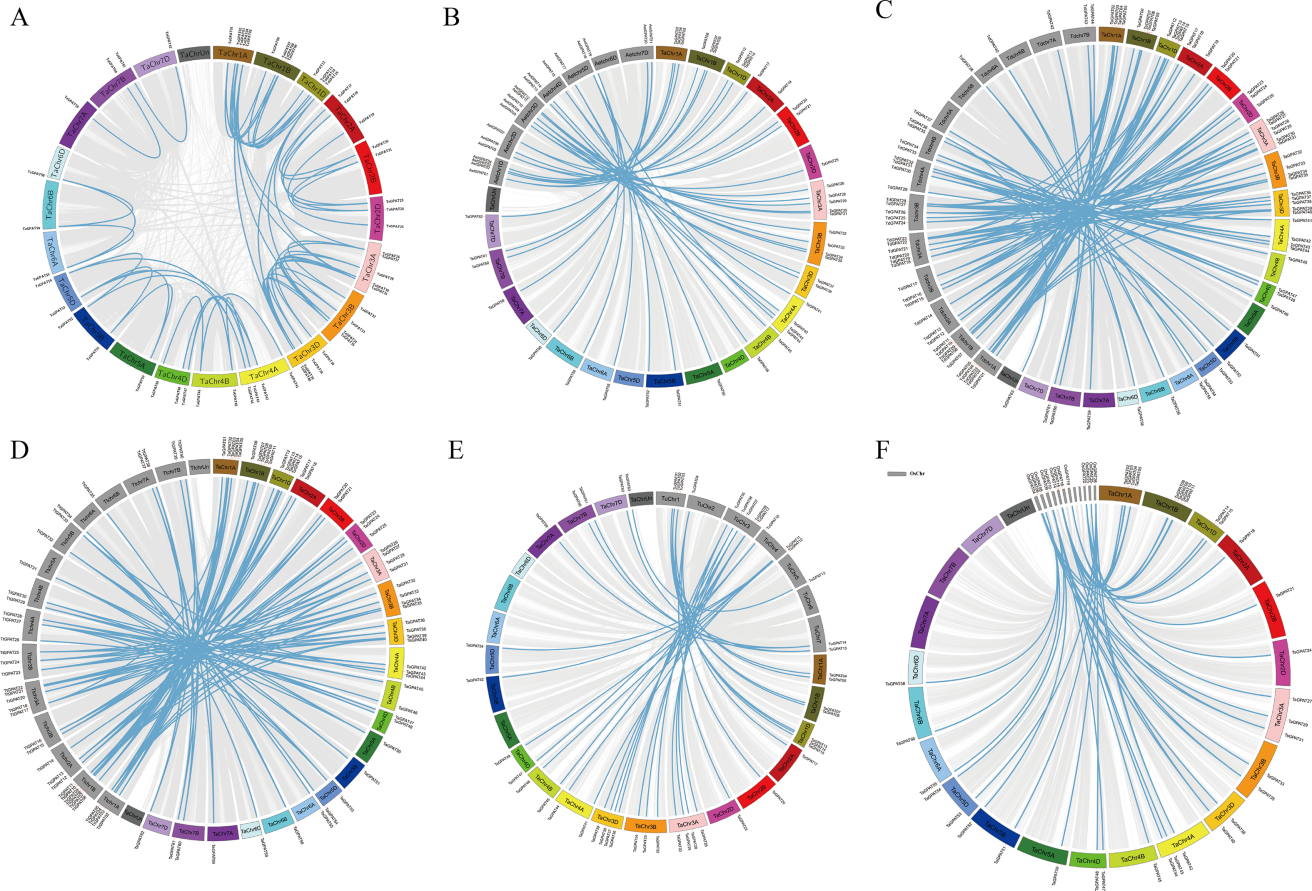
Synteny analysis of *TaGPATs* in wheat and related species. **(A)** Syntenic relationships of GPAT genes within the wheat genome. **(B–F)** Collinearity analysis between wheat and related species, including *Ae. tauschii***(B)**, *T. dicoccoides***(C)**, *T. turgidum***(D)**, *T. urartu***(E)**, and *Oryza sativa***(F)**. Chromosomes are shown in different colors. Blue lines represent syntenic gene pairs of *GPAT* genes, while gray lines indicate syntenic blocks connecting the corresponding chromosomes.

Gene duplication is a key driving force for the expansion and functional diversification of gene families. This study conducted a genome-wide identification of duplication events and analyzed the synteny to elucidate the evolutionary forces of the *TaGPAT* family. A total of 47 duplicated gene pairs were identified among the *TaGPATs*, and they were all attributed to segmental duplication, while no tandem duplication events were identified (**Figure 3A**; **Supplementary Table 3**). This indicates that segmental duplication is the predominant mechanism that drove the expansion of this gene family. To further explore the influence of natural selection on the *TaGPAT* genes, the ratios of nonsynonymous (Ka) to synonymous (Ks) substitution rates were calculated. Based on alignments of CDS and protein sequences, all gene pairs exhibited Ka/Ks ratios were < 1. The use of the Ks values in conjunction with a molecular clock model enabled an estimation of the divergence time of these duplicated gene pairs of approximately 15 million years ago (Mya) on average.

The phylogenetic history of the *GPAT* gene family was determined by conducting synteny analyses among common wheat and its four progenitor species, as well as rice (**Figures 3B–F**; **Supplementary Tables 4**-**8**). A total of 40, 86, 77, 36, and 40 duplicated gene pairs were identified in these comparative analyses. The Ka/Ks ratios of all the duplicated gene pairs were < 1. The analysis of the time of divergence of syntenic gene pairs revealed the evolutionary history of the *GPAT* gene family in the Poaceae species. The results showed that the times of divergence between common wheat and *Ae. tauschii*, *T. dicoccoides*, *T. turgidum*, and *T. urartu* were highly similar and estimated at approximately 14, 13, 13, and 11 Mya, respectively. In contrast, the time of divergence between common wheat and rice was estimated at approximately 31 Mya. These findings suggest that the evolutionary timing of the *GPAT* gene family is highly consistent with the phylogenetic relationships among these species. The divergence event between common wheat and rice has the oldest time of divergence, whereas the divergences between common wheat and its progenitor species occurred more recently.

### 3.5 Analysis of the *TaGPAT* gene promoters

The *cis*-acting elements located within gene promoter regions are key regulatory structures that control the expression of genes. To identify such elements, the 2,000 bp upstream sequences of *TaGPAT* genes were extracted as putative promoter regions. They were then submitted to the PlantCARE database for an analysis of the *cis*-elements (**Supplementary Figure 2**; **Supplementary Tables 9**, **10**). A total of 14 specific *cis*-acting elements were identified, which were classified into the following four major categories: elements responsive to hormones, environmental stress, light, and those specific for the development of tissue. The hormone-responsive elements accounted for approximately 50% of the total, including methyl jasmonate (MeJA)-responsive elements, abscisic acid (ABA)-responsive elements, salicylic acid (SA)-responsive elements, auxin-responsive elements, and gibberellin-responsive elements. Notably, the MeJA- and ABA-responsive elements were the most abundant and present in multiple copies in almost all the *TaGPAT* gene promoters. Additionally, the other three hormone-responsive elements were widely distributed and occurred in more than 50% of the *TaGPAT* promoter regions. Light-responsive elements were also present in multiple copies across all the *TaGPAT* promoters, including elements, such as the G-box and Sp1. These findings suggest that hormone-responsive and light-responsive elements are highly conserved and represent the two most important classes of *cis*-elements that regulate the transcription of *TaGPAT*. Furthermore, several genes contained four types of environmental stress-responsive elements, including elements responsive to defense and stress, those responsive to low temperature, and a MYB binding site involved in the inducibility of drought, and essential for the anaerobic induction element. These findings suggest that *TaGPAT* may play a significant role in the response to abiotic stress. Two types of tissue development-specific elements – endosperm expression regulatory elements and meristem expression elements – were also present in the promoters of most genes. This indicates that the *TaGPAT* genes play indispensable roles at multiple stages of plant development.

### 3.6 Gene ontology annotation analysis of the *TaGPATs*

**Figure 4:**
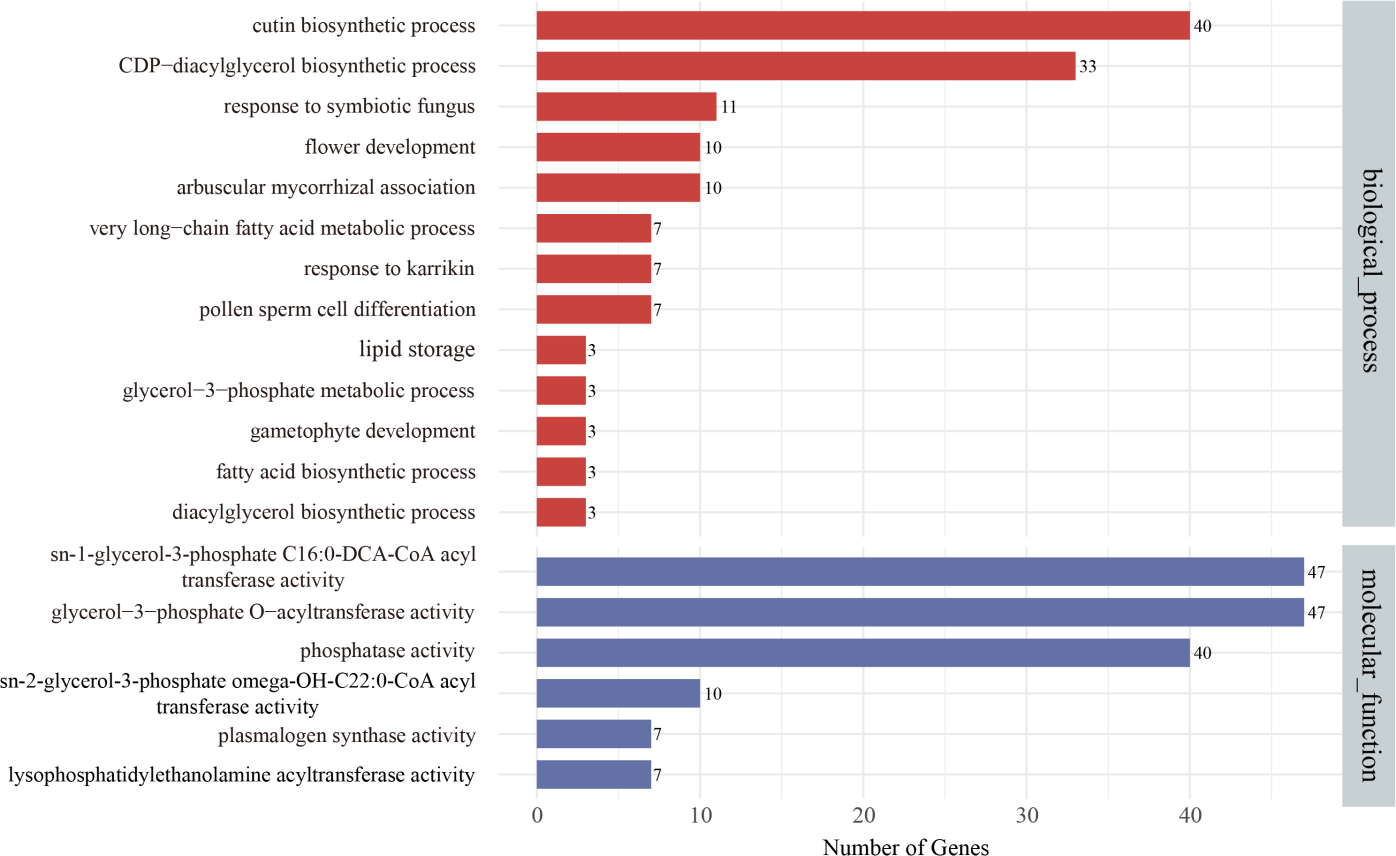
Gene Ontology (GO) annotation of *TaGPATs*. *TaGPATs* were annotated with GO terms classified into biological process (BP) and molecular function (MF) categories. The x-axis shows the number of genes annotated to each GO term, and the y-axis lists the corresponding GO terms.

The functional characteristics of the *TaGPAT* genes were analyzed in more detail by conducting a GO annotation for all 64 *TaGPATs* (**Figure 4**; **Supplementary Table 11**). The results showed that all of the genes were annotated to 19 GO terms within the biological process and molecular function categories. A total of 13 GO terms were closely associated with lipid metabolism, and three terms were related to the reproductive development of plants. Within the biological process category, 40 and 33 genes were enriched in the cutin biosynthetic process and CDP-diacylglycerol biosynthetic process pathways, respectively, which was markedly higher than for the other terms. These findings suggest that the *TaGPAT* genes may play pivotal roles in these pathways. In addition, 10, 7, and 3 genes were enriched in flower development, pollen sperm cell differentiation, and gametophyte development terms, respectively, which suggests their potential involvement in the processes of reproduction. In the molecular function category, 47 genes were annotated with *sn*-1-glycerol-3-phosphate C16:0-DCA-CoA acyltransferase activity and glycerol-3-phosphate O-acyltransferase activity, while 40 genes were enriched in the phosphatase activity category. These results collectively indicate that TaGPAT plays a key role in the enzyme activities related to lipid metabolism.

### 3.7 Expression patterns of *TaGPATs* in different tissues

**Figure 5:**
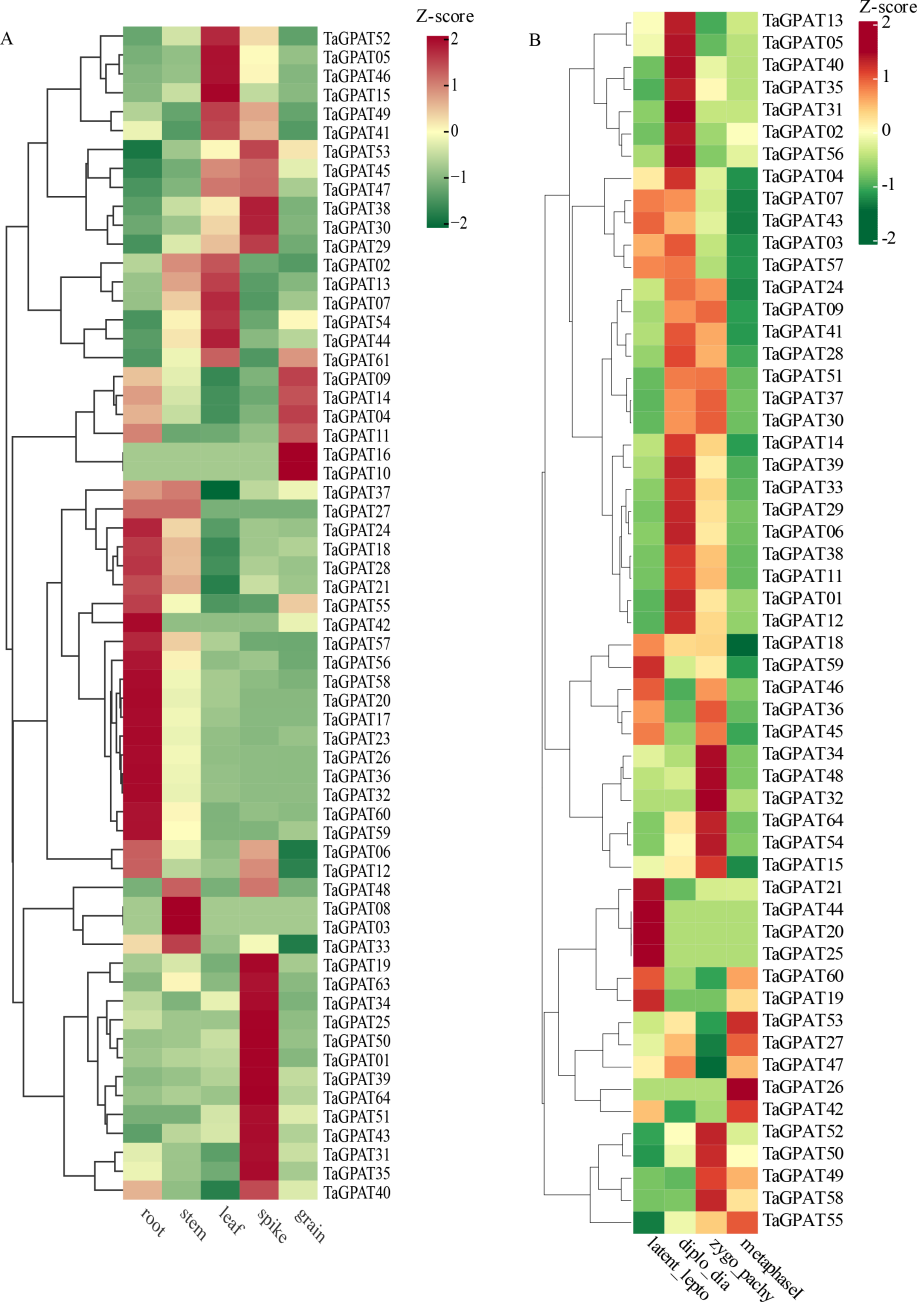
Expression patterns of *TaGPATs* in different tissues and meiotic stages. **(A)** Heatmap showing *TaGPATs* expression levels in five wheat tissues: root, stem, leaf, spike, and grain. **(B)** Heatmap illustrating expression patterns of *TaGPATs* during four meiotic stages: latent_lepto, diplo_dia, zygo_pachy, and metaphase I. Expression values were normalized using Z-score transformation.

Based on the Transcripts Per Million (TPM) values obtained from the WheatOmics database, this study analyzed the tissue-specific expression patterns of *TaGPATs* in the roots, stems, leaves, spikes, and grains of wheat (**Figure 5A**). The results revealed a substantial divergence in expression among the five tissues. A total of 21 genes were highly expressed in the roots, but they were poorly expressed in the leaves and spikes. This suggests that these genes may function specifically in the root. Only seven genes were highly expressed in the grains, which suggests that these *TaGPATs* may play more critical roles in the development of grain and accumulation of energy than the other family members. A total of 19 genes were highly expressed in the spikes. A total of 13 of the genes were specifically highly expressed in the spikes, while the remaining six genes were highly expressed in both the spikes and leaves. This pattern of expression suggests that these genes may be functionally involved in both tissues. The distinct preferential expression of the different *TaGPATs* in particular tissues further supports the functional diversification within this gene family. In addition, the dynamic expression of the *TaGPATs* exhibited pronounced variation across the four stages of wheat meiosis (**Figure 5B**). Only 10 *TaGPATs* were highly expressed in metaphase I, which suggests that these genes may predominantly function at this stage. Four *TaGPATs* were markedly highly expressed at the latent_lepto stage but maintained relatively low levels of expression thereafter, which suggests their potential importance in initiating meiosis. Notably, more than 20 genes showed high levels of expression during two critical stages of meiosis (diplo_dia and zygo_pachy). This stage-specific high level of expression indicates that the *GPAT* genes play an important role in the core biological process of meiosis.

### 3.8 Expression patterns analysis of *TaGPATs* in anthers

**Figure 6:**
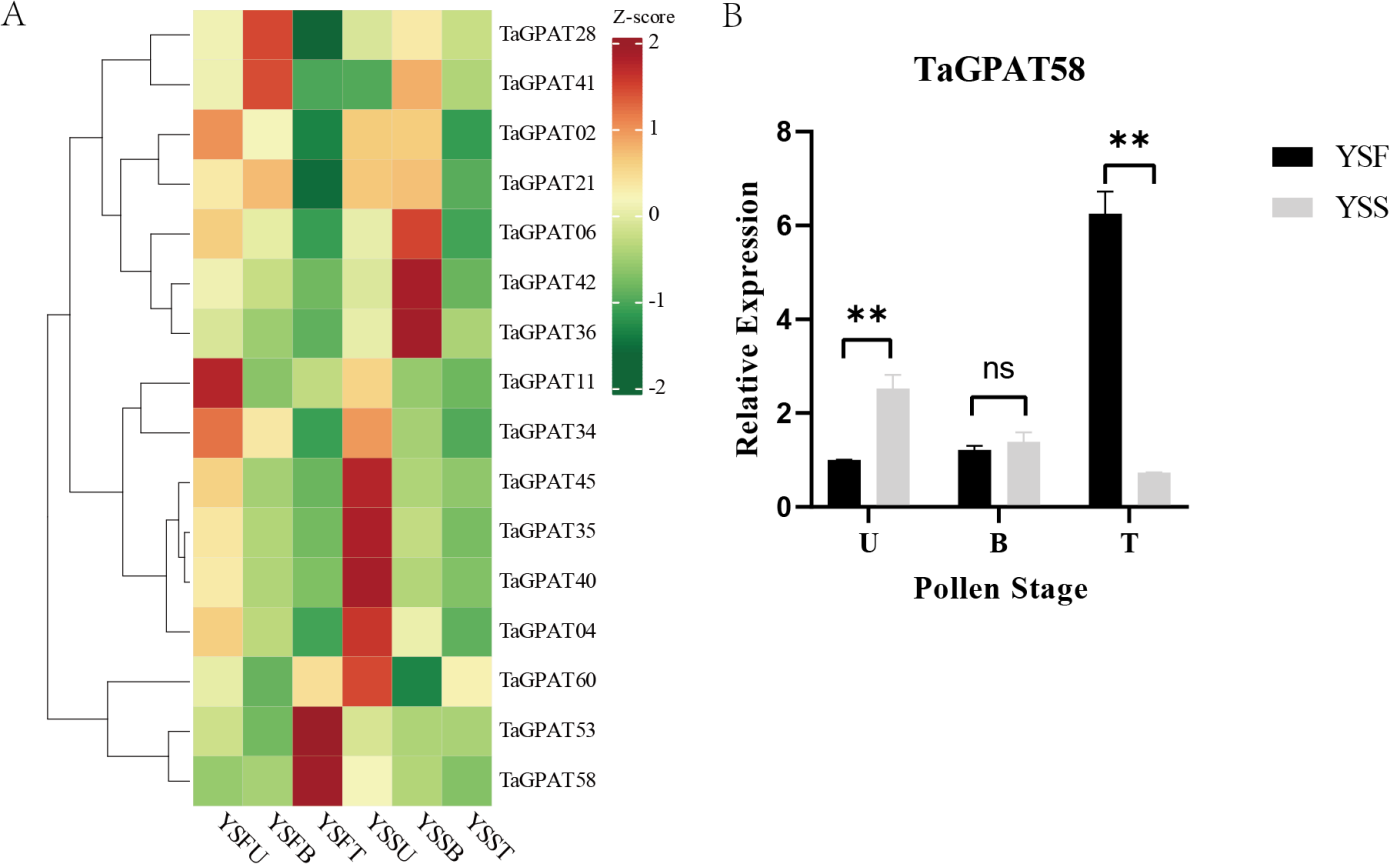
Expression of *TaGPATs* in wheat anthers under fertile and sterile conditions by qRT-PCR. **(A)** Heatmap showing relative expression levels of *TaGPATs* across different pollen developmental stages in fertile and sterile anthers. **(B)** Relative expression levels of *TaGPAT58* at various pollen developmental stages. The x-axis represents pollen developmental stages, and the y-axis shows relative expression levels. Black bars indicate fertile (YSF) samples, while gray bars indicate sterile (YSS) samples. “**” denote statistically significant differences between fertile and sterile conditions (Student’s t-test, *P* < 0.01).

The relationship between *TaGPATs* and fertility conversion was explored in the thermo-sensitive male sterile wheat line YS3038 by examining the expression patterns of 16 *TaGPATs* using qRT-PCR in anthers at the uninucleate (U), binucleate (B), and trinucleate (T) stages under both fertile (YSF) and sterile (YSS) conditions (**Figure 6A**; **Supplementary Table 12**). *TaGPAT28* and *TaGPAT41* exhibited a similar trend of first increasing and then decreasing under both conditions. They had slightly lower levels of expression under sterility during the binucleate stage. However, this difference was not statistically significant. *TaGPAT06*, *TaGPAT42*, and *TaGPAT36* were expressed at markedly higher levels during the binucleate stage under sterility, which may be associated with metabolic disorders during the development of anthers in sterile conditions. In addition, five genes (*TaGPAT45*, *TaGPAT35*, *TaGPAT40*, *TaGPAT04*, and *TaGPAT60*) were significantly more expressed at the uninucleate stage under sterility than under fertility, and such abnormal expression patterns may result from improper developmental signaling, which can lead to the premature activation of anther development. Notably, there were pronounced differences in the expression of *TaGPAT53* and *TaGPAT58* at the trinucleate stage between fertile and sterile conditions. However, that transcriptomic data from this study (unpublished data) indicated no obvious difference for *TaGPAT53* at this stage. In contrast, the level of expression of *TaGPAT58* in the fertile anthers at the trinucleate stage was approximately six-fold higher than that under sterility (**Figure 6B**), which was also consistent with the transcriptomic results (**Supplementary Figure 3**). Therefore, this study proposes that *TaGPAT58* plays a crucial role in the development of anthers at the trinucleate stage.

### 3.9 The roles of *TaGPAT58* and its homologous genes in the development of pollen in wheat

**Figure 7:**
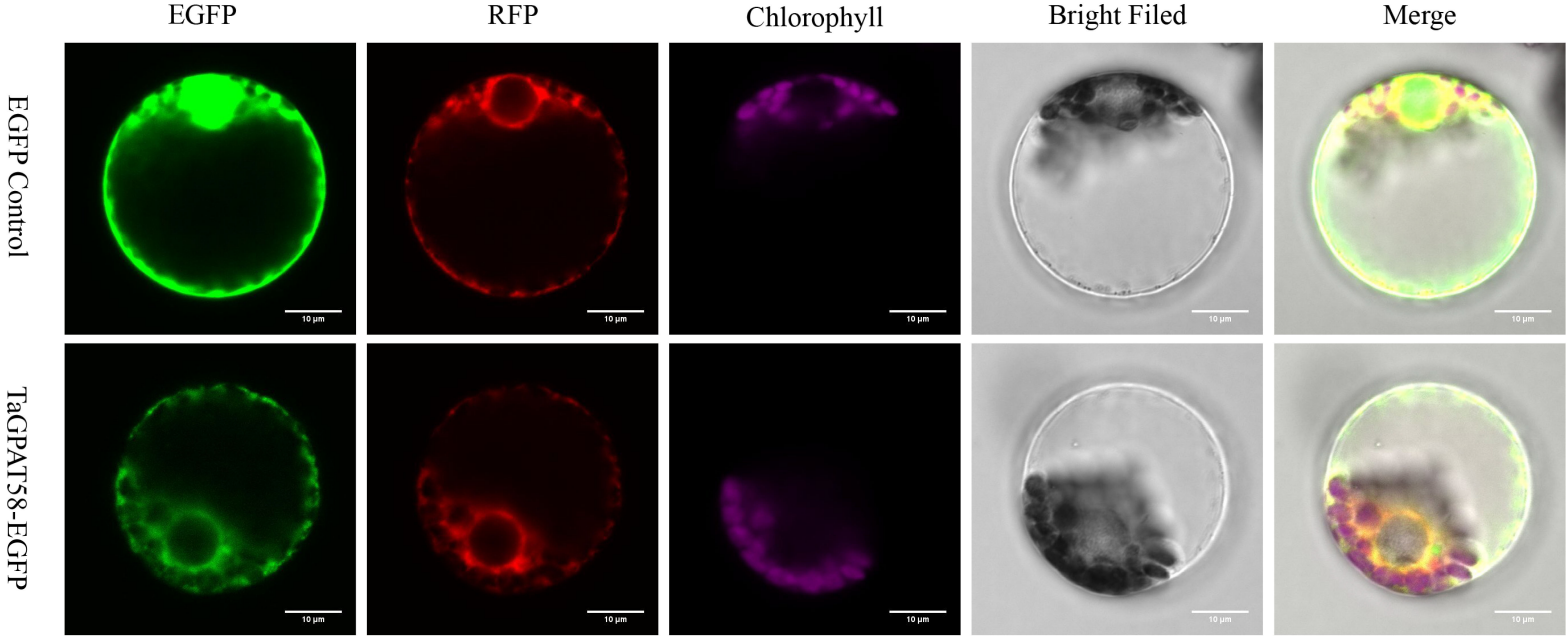
Subcellular localization of TaGPAT58 in wheat protoplasts. Confocal images showing the subcellular localization of TaGPAT58 in wheat protoplasts. The upper row shows the empty vector control, and the lower row shows the TaGPAT58–EGFP fusion construct. From left to right: EGFP fluorescence, RFP fluorescence indicating the (ER) marker, chlorophyll autofluorescence, bright‐field image, and merged image. The EGFP signals from the TaGPAT58 fusion protein co-localize with the ER marker, indicating that TaGPAT58 is localized to the endoplasmic reticulum. Scale bars = 10 μm.

To clarify the subcellular localization of the TaGPAT58 protein and better elucidate its biological function. This study constructed a pCAMBIA1302-*TaGPAT58*-EGFP fusion expression vector, and pCAMBIA1302-EGFP served as a control. The constructs were transiently transformed into wheat protoplasts to observe their fluorescence. The results showed that the signal for the TaGPAT58-EGFP fusion protein fluorescence highly overlapped with an ER marker protein, while no overlap was observed with the chloroplast autofluorescence (**Figure 7**). This indicated that TaGPAT58 is localized to the ER.

The role of *GPAT* genes in male reproductive development in wheat was studied further by utilizing barley stripe mosaic virus–mediated virus-induced gene silencing (BSMV-VIGS) to analyze their function. Four treatment groups exhibited corresponding phenotypes after infection (**Supplementary Figure 4**). The leaves of the control group had no stripes, which would be a symptom of disease. In contrast, the other three groups developed typical striped lesions, and the leaves of the positive control bleached. Similar bleaching was observed in the spikes of the positive control. These results confirmed the efficacy of the VIGS system in this experiment.

**Figure 8:**
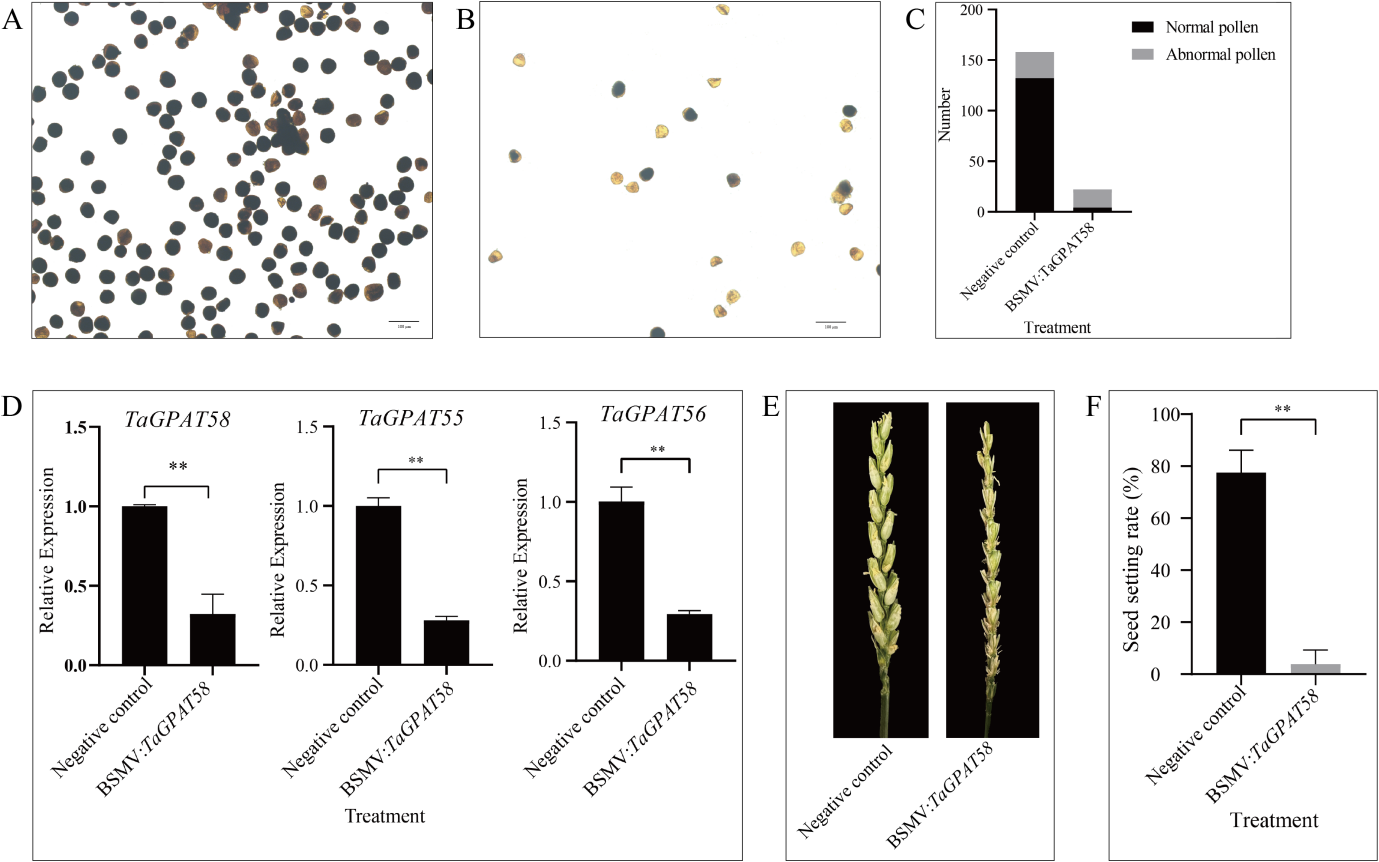
Effects of *TaGPAT58* silencing on pollen development, seed setting, and gene expression in wheat. I_2_–KI staining of trinucleate pollen showing normal viable pollen in the negative control **(A)** and significantly reduced pollen viability in *TaGPAT58*-silenced plants **(B)**. **(C)** Quantification of normal and abnormal pollen grains in negative control and *TaGPAT58*-silenced plants. **(D)** Relative expression levels of *TaGPAT58* and its two homologous genes, *TaGPAT55* and *TaGPAT56*, in trinucleate-stage anthers of negative control and silenced plants, as determined by qRT-PCR. **(E)** Seed-setting phenotypes of negative control and *TaGPAT58*-silenced plants. **(F)** Statistical analysis of seed-setting rate in negative control and silenced plants. “**” indicates extremely significant differences (Student’s t-test, *P* < 0.01).

At the trinucleate stage of anther development, the anthers from the negative control and *TaGPAT58*-silenced plants were stained with iodine-potassium iodide (I_2_-KI) (**Figures 8A, B**). Microscopic observations showed that pollen grains from the negative control plants were uniformly stained, regularly shaped, and structurally intact, whereas the pollen grains in the gene-silenced plants stained less intensely, had an irregular morphology, and partially collapsed. Pollen counting showed that the negative control contained a total of 158 pollen grains, 26 of them were lightly stained or morphologically abnormal, this corresponded to an abnormal pollen rate of 16.46%. In contrast, the gene-silenced plants produced a total of only 22 pollen grains, and 18 were abnormal, thus resulting in an abnormally high abnormal pollen rate of 81.82% (**Figure 8C**). These results indicate that the development of pollen was severely impaired in the gene-silenced plants, with a marked reduction in pollen number and a significantly higher proportion of abnormal pollen compared with the negative control. Subsequently, the expression level of *TaGPAT58* in anthers at the trinucleate stage was examined in both treatments (**Figure 8D**). The results showed that the expression of *TaGPAT58* was significantly reduced in the silenced plants compared with the negative control. Notably, *TaGPAT58* has a highly similar sequence to its two homologs, *TaGPAT55* and *TaGPAT56*, with an overall sequence identity of 96.76%; the identity within the VIGS-targeted region reached 97.65% (**Supplementary Figure 5**). Therefore, the expression levels of *TaGPAT55* and *TaGPAT56* were also significantly reduced (**Figure 8D**). A significant difference in the seed-setting rate was observed between the two treatments. Nine spikes were examined and analyzed statistically for each treatment (**Figures 8E, F**; **Supplementary Table 13**). The negative control plants produced relatively plump grains, with a seed-setting rate of approximately 78%, whereas the *TaGPAT58*-silenced plants were almost completely sterile. Their seed-setting rate was only 0.04%. Taken together, these results demonstrate that *TaGPAT58* and its homologous genes are indispensable for the development of normal pollen and the maintenance of fertility in wheat.

## 4 Discussion

### 4.1 Characteristics of the wheat *GPAT* gene family

The core feature of the *GPAT* gene family is that all its members contain the acyltransferase domain (PF01553). This family plays important roles in plant growth, development, and responses to abiotic stresses. The *GPAT* gene family has been identified in various plant species, including 10, 26, 20, and 22 members in *A. thaliana* (Waschburger et al., 2018), rice (Safder et al., 2021), maize (Zhu et al., 2019), and barley (Yang et al., 2024), respectively. Although wheat is an important cereal crop, the *GPAT* gene family in it had not yet been systematically characterized. In this study, we utilized a combined strategy using HMM and BlastP to identify 64 *TaGPATs* in common wheat. Additionally, 21 *AetGPATs*, 44 *TdGPATs*, 41 *TtGPATs*, and 17 *TuGPATs* were identified in its progenitor species. The number of *GPAT* genes in common wheat is significantly higher than in its diploid and tetraploid progenitors. There was a gradual increase with increasing ploidy level, which is highly consistent with the evolutionary history of common wheat. The *GPAT* gene family markedly expanded during the evolution of wheat. This expansion was primarily driven by whole-genome duplication (WGD) events. The high retention rate of the family members following WGD suggests that this gene family plays important roles in the growth and development of wheat. Notably, this pattern of expansion is consistent with previous observations in wheat gene families, such as the *E-class* (Bai et al., 2024) and *TPS* (Liu et al., 2024), and their copy numbers also increase with ploidy level, which further confirms the central role of WGD in the expansion of gene families in wheat. Compared with diploid species such as *A. thaliana*, rice, maize, barley, and wheat possess the largest number of *GPAT* family members. This is primarily because wheat is an allohexaploid (AABBDD) species. During WGD, it retained a large number of homologous genes from its progenitor species, and subsequent gene duplication events further promoted the expansion of the GPAT gene family in wheat (Zhu et al., 2020). Significant variation was observed in the length of amino acids, MW, pI, and subcellular localization among the TaGPAT proteins. This indicated that while the conserved domains of this family are preserved, functional divergence has occurred among the family members to some extent (Goldtzvik et al., 2023).

### 4.2 Evolutionary analysis of the *TaGPATs*

The phylogenetic relationships of the wheat *GPAT* gene family was comprehensively analyzed by constructing three types of phylogenetic trees. The first was an intra-species phylogenetic tree that contained only wheat *GPAT* family members was constructed to elucidate the internal structure and characteristics of the classification of this family within wheat. Secondly, a cross-species phylogenetic tree based on GPAT protein sequences from wheat, maize, and *A. thaliana* was generated to compare the phylogenetic relationships and evolutionary divergence patterns of the *GPAT* genes between the monocotyledonous and dicotyledonous plants. Third, a phylogenetic tree that integrated the GPAT protein sequences from wheat and its progenitor species was constructed to trace the evolutionary origins and trajectories of the *TaGPAT* gene family during the evolution of wheat. This multi-dimensional phylogenetic analysis provides a solid foundation to understand the expansion, divergence, and conservation of the *GPAT* genes in wheat (Rawal et al., 2013). The phylogenetic analysis revealed that all the trees could be divided into three clades, and clade III was split into two subclades. *AtS1* and *AtGPAT09* were members of clades I and II, respectively, which contained 3 and 10 *TaGPATs*. The remaining *AtGPAT* genes clustered within the two subclades of clade III, which also included most of the *TaGPAT* genes. This grouping pattern is generally consistent with previous studies in other species of plants (Zhu et al., 2020). In the phylogenetic tree that involves wheat and its progenitor species, all three clades contained *GPAT* genes from all five species. This indicates that the family is largely intact across these lineages without obvious gene loss.

The analysis of the structures of genes revealed further differences among the clades. The 13 *TaGPAT*s in clades I and II contained fewer motifs but exhibited more complex gene structures with 12–13 exons, which sharply contrasted with the gene structures in clade III. This structural divergence suggests that the genes in clades I and II may perform specific functions. Notably, this structural pattern is highly consistent with the observation in distantly related species, such as bean and caper spurge (*Euphorbia lathyris* L.) (Kasapoglu et al., 2024; Wang et al., 2024). This study indicated that despite the structural differences among the clades, the internal structural features within each clade are highly conserved across species. This reflects the conserved organizational mode of the *GPAT* gene family throughout long-term evolution. Further synteny analysis showed that the divergence time of the syntenic gene pairs in clades I and II of wheat and its progenitor species was generally more recent than that of the clade III members (**Supplementary Table 3**). Based on the gene structure and characteristics of divergence time, it can be inferred that the genes in clades I and II may have undergone stronger functional constraints during evolution, thereby maintaining more conserved structures and potential functions.

The phylogenetic analysis across the species further revealed that all three clades contained *GPAT* genes from wheat, maize, and *A. thaliana*, indicating that core members of the *GPAT* gene family diverged before the monocot-dicot split and have conserved gene structures across diverse plant species. This observation is consistent with previous studies on the plant *GPAT* gene family (Waschburger et al., 2018). Moreover, the recent genome-wide analyses of the *GPAT* family in alfalfa support the concept that the *GPAT* genes exhibit stable structural organization and conserved phylogenetic patterns within dicots (Ma et al., 2024). This underscored the critical role of this gene family in growth and adaptation to the environment. Paralogous gene pairs were identified in all three species, which indicated a history of extensive gene duplication events during the evolution of the *GPAT* family. Common wheat, as an allohexaploid species that has undergone multiple rounds of WGD, contains the largest number of paralogous gene pairs (22 pairs) among the three species. These results are consistent with broader evolutionary studies on plant gene families, which demonstrate that metabolic enzyme gene families often undergo extensive expansion. This expansion resulted in larger family sizes and an increased potential for functional diversification (Fang et al., 2022).

In the phylogenetic tree constructed from common wheat and its four progenitor species, the *GPAT* gene family exhibits typical differentiation corresponding to the A, B, and D genomes. Orthologous gene pairs were identified as 11, 14, and 6 between wheat and *Ae. tauschii* (DD), *T. dicoccoides* (AABB), and *Triticum_turgidum* (AABB), respectively, indicating a high degree of inheritance and the retention of *GPAT* genes in these genomes. This distribution pattern is highly consistent with the evolutionary history of common wheat: *Ae. tauschii*, as the direct donor of the D genome, shares the shortest evolutionary distance with wheat, thus, retaining more one-to-one homologous relationships within the *GPAT* family. *T. dicoccoides* and *T. turgidum*, as the primary donors of the A and B genomes in modern wheat, also maintain high sequence similarity and phylogenetic clustering relationships. In contrast, no clear orthologous gene pairs were detected between wheat and *T. urartu* (AA). This phenomenon may be attributed to two reasons. First, the A genome of common wheat evolved through two rounds of distant hybridization (Marcussen et al., 2014), during which complex genome reshaping, gene duplication, and structural variations probably occurred. As a result, *TaGPAT* genes derived from the A subgenome clustered further away from *TuGPATs*. Secondly, in addition to WGD, the *TaGPATs* may also have been influenced by local duplication events, such as segmental duplication and transposon-mediated duplication during evolution, which lead to the redistribution of some gene copies within the genome and weakened their homologous relationships with the *TuGPATs*.

### 4.3 Duplication of the *GPAT* genes in wheat

Duplicated genes are widespread in plant genomes and play an essential role in the diversification of new functions, such as the development of floral organs and the adaptation to biotic and abiotic stresses (Panchy et al., 2016). Gene duplication is a major driving force for the expansion of gene families and the emergence of novel gene functions (Birchler and Yang, 2022), which primarily occurs through tandem duplication, segmental duplication, transposition events, and WGD (Flagel and Wendel, 2009; Freeling, 2009; Wang et al., 2015; Zhang et al., 2013). This study systematically analyzed the duplication events of *GPAT* genes within wheat and further compared the duplication and conservation patterns between wheat and its four progenitor species, as well as rice, to elucidate the expansion mechanism of the *GPAT* gene family. A total of 47 duplicated gene pairs were identified within wheat, while 40, 86, 77, 36, and 40 duplicated gene pairs were detected between wheat and each of its four progenitor species and rice, respectively. All of these events involved segmental duplication. This indicated that in addition to the whole-genome duplication, local segmental duplication also substantially contributed to the further expansion of the *GPAT* gene family in wheat. Previous studies have demonstrated that segmental duplication and chromosomal translocation facilitate rapid environmental adaptation in plants (Fraser et al., 2005). Therefore, this feature may partly reflect the adaptive potential of common wheat during long-term evolution and provide important clues to understand the molecular basis that underlies its broad environmental adaptability.

The Ka/Ks ratio is a widely used parameter in evolutionary studies, and it reflects the rates of nonsynonymous (Ka) and synonymous (Ks) substitutions and thus, provides insights into the selective pressures that act on genes. Generally, Ka/Ks > 1 indicates positive selection, whereas Ka/Ks < 1 suggests purifying selection (Li et al., 2025). In addition, divergence time can be estimated based on the Ks values (Gaut et al., 1996). In this study, all duplicated gene pairs exhibited Ka/Ks < 1, suggesting that the *GPAT* gene family has experienced strong purifying selection throughout the evolutionary history from diploid progenitor species to hexaploid common wheat, as well as during its divergence from the distantly related species rice, thereby maintaining structural and functional conservation of these genes (Xu and Feng, 2025). Furthermore, the estimation of the divergence time revealed that the divergence between wheat and its progenitor species occurred much more recently than that between wheat and rice, which is highly consistent with the phylogenetic relationships between the plants and the general evolutionary pattern of species divergence.

### 4.4 Expression patterns of *TaGPATs* and a functional analysis of *TaGPAT58*

Variations in the types and distributions of *cis*-acting elements in promoter regions are important contributors to the diversification of gene regulation and functional differentiation (Hernandez-Garcia and Finer, 2014). In the promoter regions of all the *TaGPATs*, we identified four major categories of characteristic *cis*-acting elements, including elements responsive to hormones, environmental stress, and light. In addition, there are specific elements that respond to tissues and development. Elements that respond to MeJA and ABA were widely detected in the promoters of the *TaGPATs*, which suggests that these hormones may participate in the transcriptional regulation of *TaGPATs*. Similarly, multiple elements that respond to hormones have also been identified in the *GPAT* gene family of barley (Yang et al., 2024). In addition, 52 *TaGPAT* promoters contained at least one *cis*-acting element associated with the responses to abiotic stress, suggesting that these elements may regulate the expression of *GPAT* genes under stress conditions and thereby enhance plant adaptability to adverse environments. Consistent with this finding, similar results have also been reported from a promoter analysis of the rice *GPAT* gene family (Safder et al., 2021).

It is generally believed that gene function depends on its spatiotemporal expression characteristics, and the expression patterns of the genes across different tissues, developmental stages, and environmental conditions provide important evidence to understand their potential biological functions (Ma et al., 2025). This study analyzed the expression levels of *TaGPATs* using transcriptome data from the WheatOmics database. This enabled study of their levels of expression in the wheat roots, stems, leaves, spikes, and grains, as well as in the anthers at four meiotic stages. The results showed that individual *TaGPATs* exhibited distinct expression patterns among different tissues and developmental stages. However, several gene clusters were only highly expressed in certain tissues or at specific developmental stages, suggesting that the different *TaGPATs* may perform diverse biological functions. Genes that were closely clustered in the phylogenetic tree tended to show similar expression patterns, such as *TaGPAT46*, *TaGPAT49*, and *TaGPAT15*, which were highly expressed in leaves, and *TaGPAT04*, *TaGPAT09*, and *TaGPAT14*, which were preferentially expressed in grains. This observation is consistent with previous findings that the duplicated genes generated by segmental duplication or WGD often display similar and functionally redundant expression patterns across multiple tissues (Almeida-Silva and van de Peer, 2025). However, during the four stages of meiosis, most homologous gene pairs exhibited markedly divergent expression patterns, indicating substantial expression divergence of *TaGPATs* during reproductive development in wheat. This suggests that they have a relatively low functional redundancy, and they may not exhibit overlapping functions across all stages of development.

Previous studies have demonstrated that the *GPAT* genes in multiple species are closely associated with male fertility in plants. In *A. thaliana*, the silencing of *AtGPAT01* leads to the severe arrest of pollen development, indicating its essential role in pollen formation (Zheng et al., 2003). The overexpression of *ElGPAT9* in tobacco (*Nicotiana benthamiana*) markedly enhances the viability of total pollen (Wang et al., 2024). In maize, the loss of function of *ZmMs33/ZmGPAT6* results in defective anther cuticles, the arrested degradation of anther wall tissues, abnormal Ubisch bodies and exine formation, ultimately causing complete male sterility (Zhu et al., 2019). In rice, the *osgpat3* mutant exhibits impaired Ubisch body formation, delayed PCD of the three inner anther wall layers, and abnormal pollen degradation. These impairments lead to the failure of pollen maturation and complete male sterility. Complementation and CRISPR/Cas9 knockout experiments confirmed that *OsGPAT3* is an essential gene for the male reproductive development in rice (Sun et al., 2018).

Even though the *GPAT* genes in multiple species have been verified to be associated with male sterility, studies on the relationship between *GPAT* genes and male fertility in wheat, a polyploid crop, remain limited. In addition, the highly homologous members of the GPAT gene family may exhibit functional redundancy. Therefore, a systematic analysis of the wheat *GPAT* gene family, investigation of their expression patterns, and integration with functional validation are highly important for elucidating their roles in the regulation of male fertility, optimizing the utilization of male-sterile lines, and guiding hybrid breeding strategies. In this study, TaGPAT58 was localized to the ER, a major site of lipid metabolism. This supports a potential role for this protein in the biosynthesis of lipids. Furthermore, the silencing of *TaGPAT58* using BSMV-VIGS resulted in a reduction in the pollen grains, abnormal pollen morphology, and a significant decrease in the rate of seed-setting. The principle of VIGS is to insert a partial sequence of the target gene into a viral vector, which is transcribed in host cells to generate double-stranded RNA (dsRNA). This dsRNA is subsequently processed by Dicer-like into short interfering RNAs (siRNAs) of approximately 21–23 nucleotides. These siRNAs are then incorporated into the RNA-induced silencing complex (RISC) and direct the degradation of transcripts that share at least 23 consecutive nucleotides of sequence identity with the target. Consequently, this technique inevitably silences homologous genes with high sequence similarity (Burch-Smith et al., 2004; Kirigia et al., 2014). In this study, *TaGPAT58* shows high sequence similarity with its two homologous genes, *TaGPAT55* and *TaGPAT56*. Therefore, siRNAs derived from the TaGPAT58-targeting fragment are able to recognize and act on the transcripts of these homologs as well, resulting in their concomitant down-regulation in silenced plants. To some extent, this coordinated silencing of homologous genes helps to reduce functional redundancy among *TaGPAT58* and its homologs, thereby providing a more comprehensive understanding of their roles in wheat male reproductive development. The phylogenetic analysis showed that *TaGPAT58* clusters with *AtGPAT04*, *AtGPAT06*, and *AtGPAT08* in clade III-2, suggests that the structure and function of these proteins are similar. Previous studies have shown that these *A. thaliana* genes possess both *sn*-2 acyltransferase and phosphatase activities, exhibit high substrate specificity toward C16 and C18 ω-hydroxy acyl-CoA, and participate in the biosynthesis of cutin (Yang et al., 2012). AtGPAT06 also plays multiple roles in pollen exine formation, tapetum development, and stamen elongation (Li et al., 2012). The GO enrichment analysis revealed that TaGPAT58 is primarily enriched in phosphatase activity, *sn*-2-glycerol-3-phosphate omega-OH-C22:0-CoA acyl transferase activity, cutin biosynthetic process, and flower development, which is consistent with the known functions of its homologs *in A. thaliana*. Taken together, these results suggest that *TaGPAT58* plays an important role in the development of the anther cuticles and pollen walls and is indispensable for male reproductive development in wheat. Although this study preliminarily analyzed the potential roles of *TaGPAT58* and its homologous genes in the development of anthers using VIGS, this approach is inherently limited because VIGS induces only transient gene silencing and cannot selectively suppress highly homologous genes, thereby constraining independent functional validation of individual genes. Moreover, the specific signaling pathways and metabolic processes regulated by *TaGPAT58* during the male reproductive development of wheat remain unclear. Therefore, systematic functional verification using stable mutant lines will be required to comprehensively elucidate the molecular mechanisms underlying the role of this gene in male reproductive development.

## 5 Conclusion

In this study, a total of 64 *TaGPATs* were identified through a genome-wide analysis and classified into three clades based on their phylogenetic relationships. An analysis of the duplication events indicated that segmental duplication was the key driving force for the wheat *GPAT* gene family expansion. The expression of the *TaGPATs* varied across different tissues and developmental stages of reproductive growth, which may be closely related to the diverse gene structures within each clade and the variation in *cis*-acting elements in their promoter regions. In the temperature-sensitive male sterile line YS3038, *TaGPAT58* expression was significantly downregulated at the trinuclear stage of pollen development under sterile conditions, which was consistent with results of transcriptome sequencing. Subcellular localization revealed that TaGPAT58 is localized to the ER. Further gene-silencing experiments revealed that silencing of *TaGPAT58* simultaneously led to reduced expression of *TaGPAT58* and its two homologous genes, *TaGPAT55* and *TaGPAT56*, in anthers. The silenced plants exhibited a marked decrease in pollen number, abnormal pollen morphology, and a significantly reduced seed-setting rate, ultimately resulting in complete male sterility. These results indicate that *TaGPAT58* and its homologous genes play critical roles in wheat pollen development.

## Data Availability

The original contributions presented in the study are included in the article/**Supplementary Material**. Further inquiries can be directed to the corresponding authors.
