# Supplementary material for: Genome-wide analysis of the GPAT gene family in wheat and the potential roles of TaGPAT58 and its homologs in male reproductive development
Source: Front Plant Sci. 2026 Feb 27;17:1779919. doi: 10.3389/fpls.2026.1779919 (PMC12982185; doi:10.3389/fpls.2026.1779919)
Supplement: Supplementary file 1 [file DataSheet1.docx]

Supplementary Figures


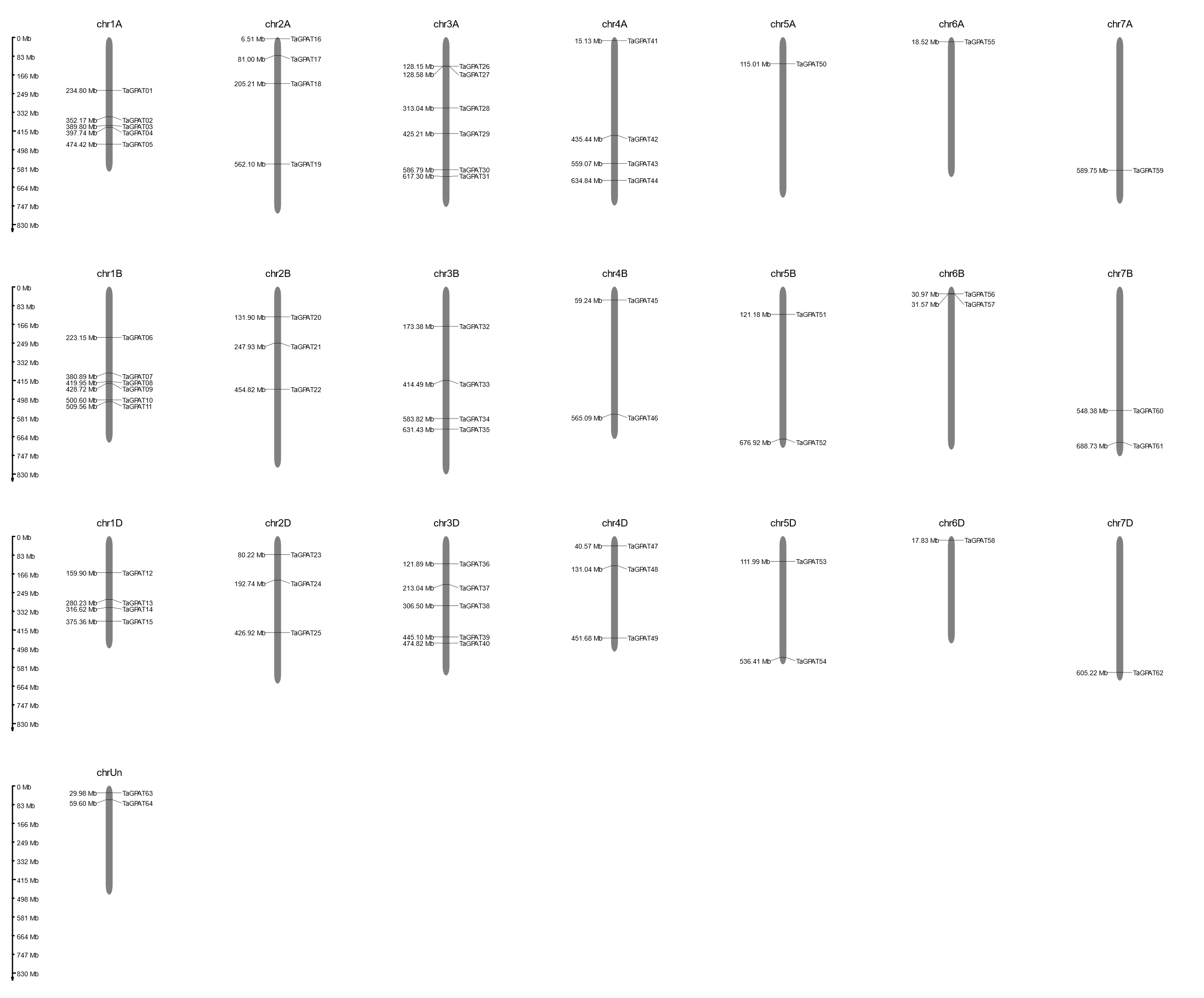


Supplementary Figure S1. Chromosomal distribution of *TaGPATs* in wheat.


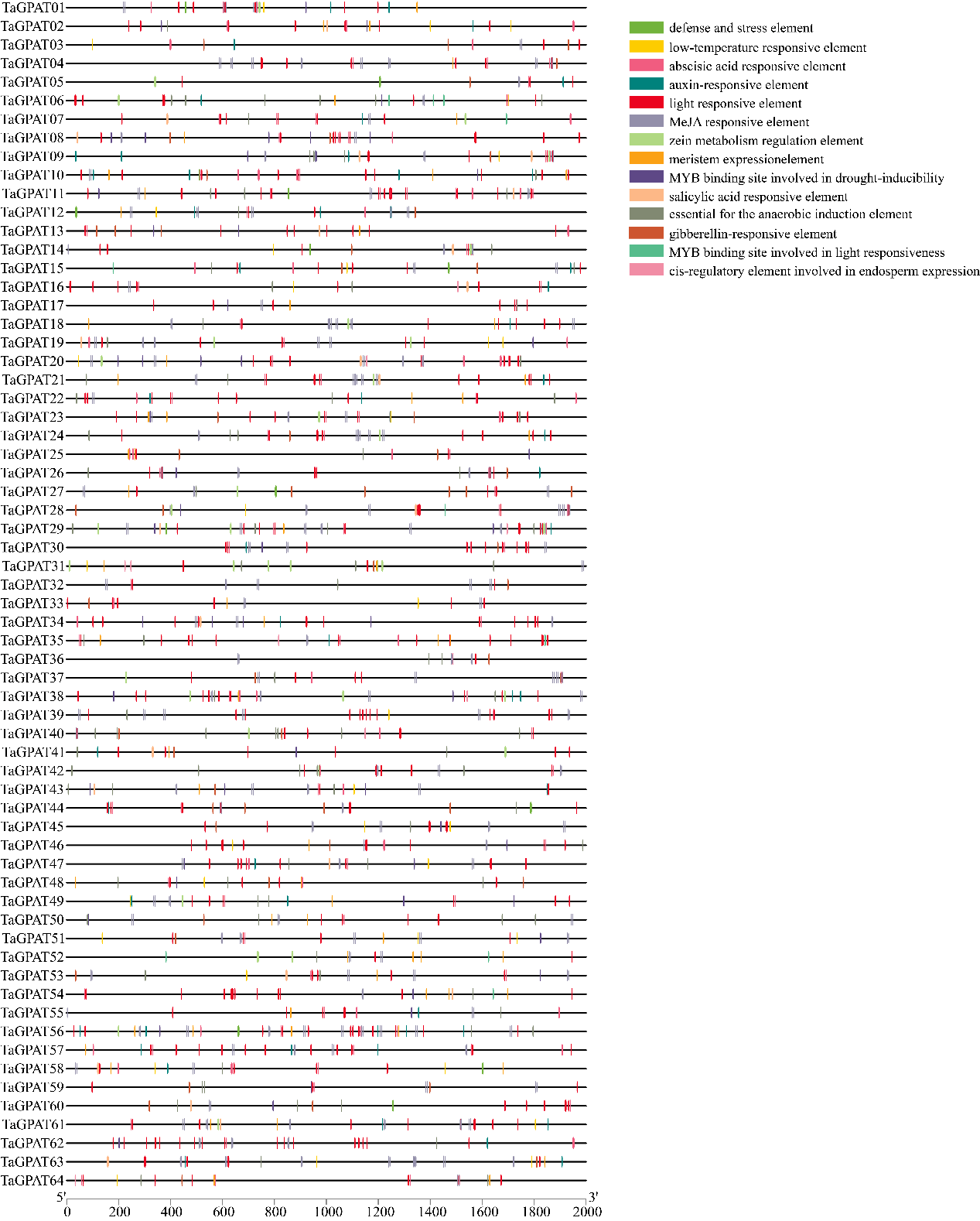


Supplementary Figure S2. Distribution of cis-acting elements in the promoter regions of *TaGPATs*. The figure illustrates the distribution of cis-acting elements within the 2000 bp upstream promoter regions of *TaGPATs*. Different colored boxs represent distinct types of cis-acting elements,


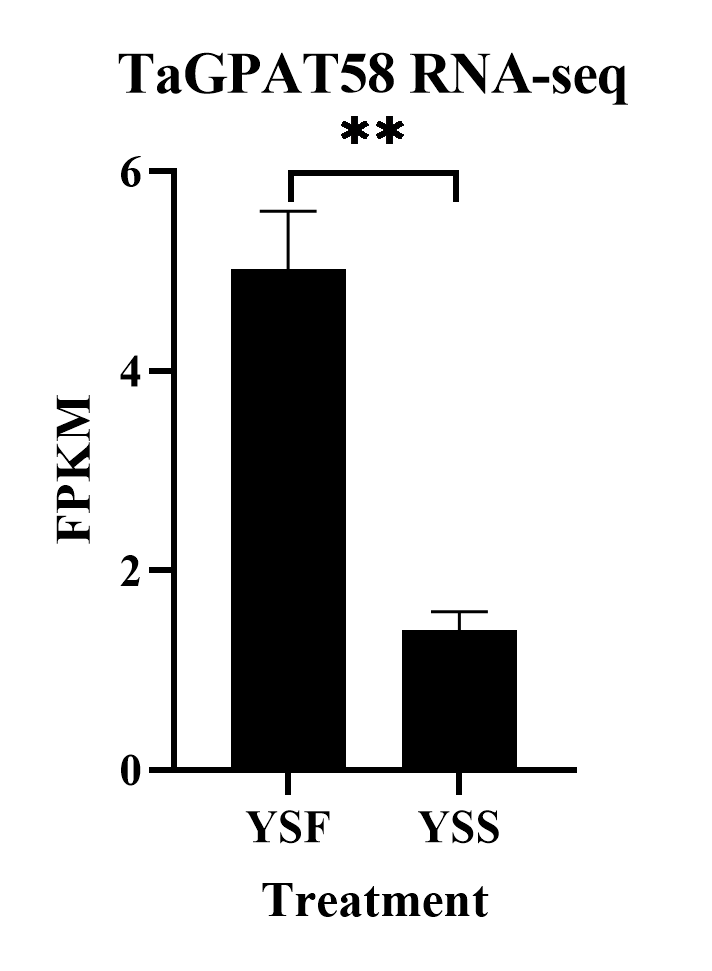


Supplementary Figure S3. Differential expression analysis of *TaGPAT58* based on FPKM values.

The figure shows the differences in FPKM expression levels of TaGPAT58 between YSF and YSS. "**" indicates extremely significant differences determined by Student’s t-test (P < 0.01).


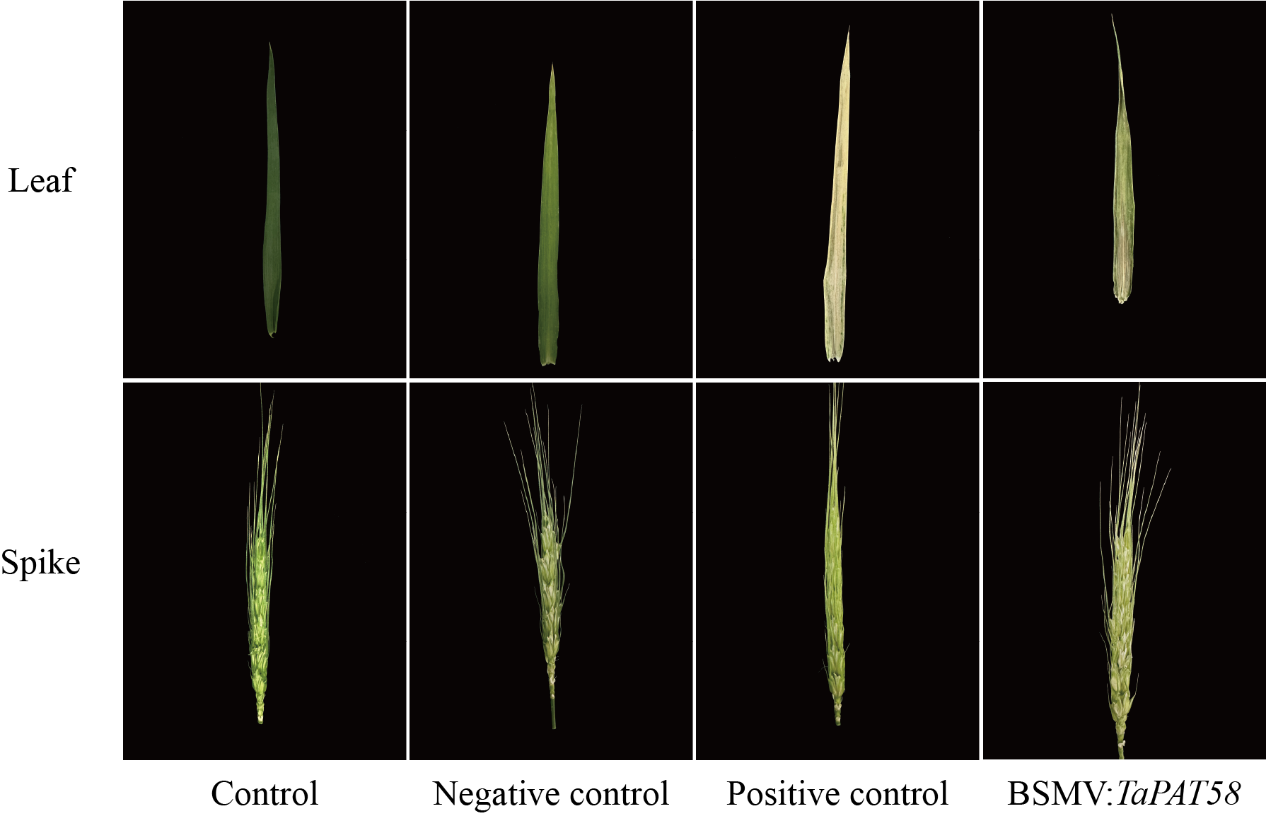


Supplementary Figure S4. Phenotypes of leaves and spikes under different treatment combinations after BSMV-VIGS.


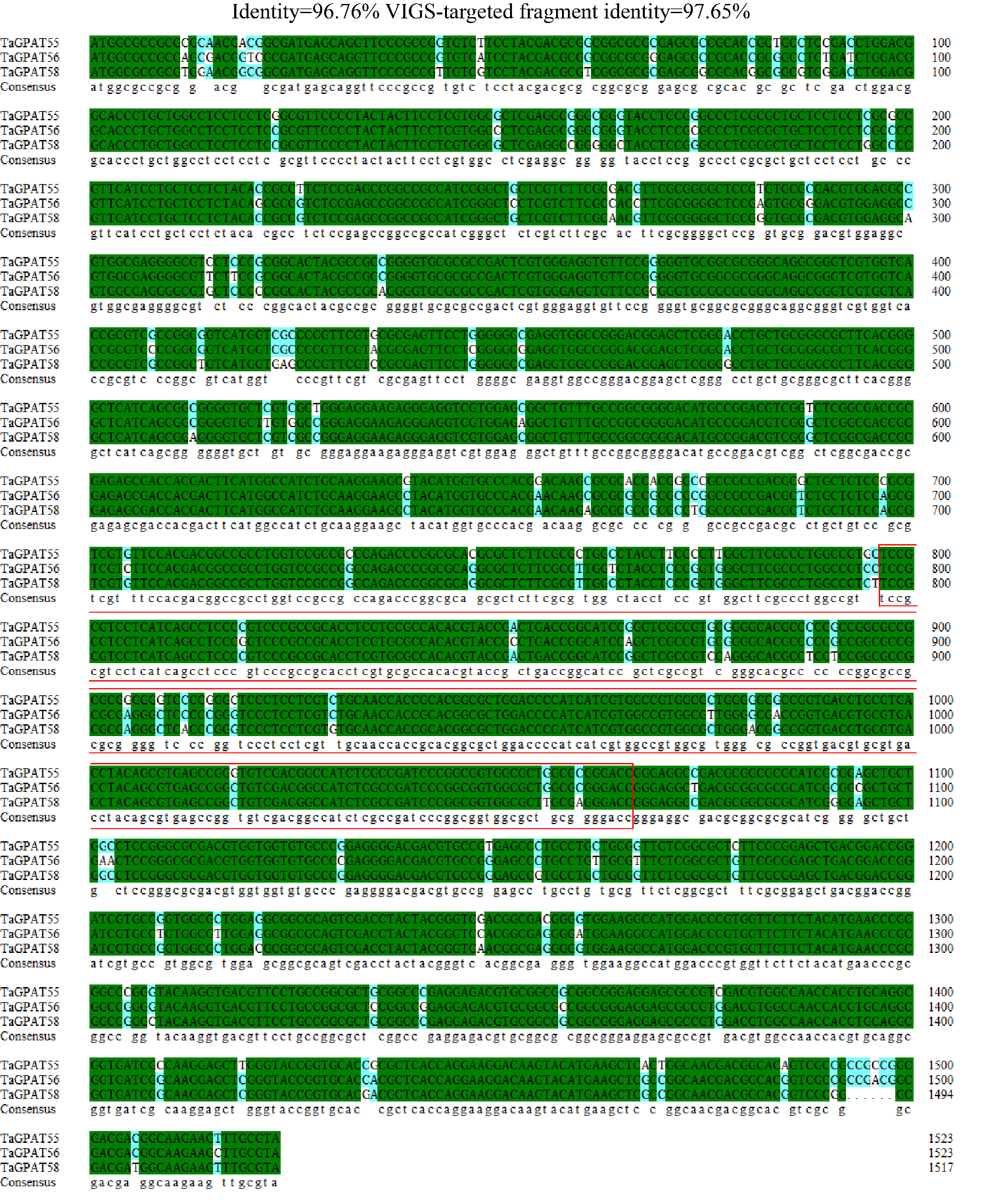


Supplementary Figure S5. Multiple sequence alignment of *TaGPAT58*, *TaGPAT55*, and *TaGPAT56*. The overall sequence identity among the three genes is 96.76%, and the VIGS-targeted fragment shows an identity of 97.65%. The VIGS-targeted fragment is highlighted with a red box.
